# Shared reference materials harmonize lipidomics across MS-based detection platforms and laboratories[S]

**DOI:** 10.1194/jlr.D119000393

**Published:** 2019-11-15

**Authors:** Alexander Triebl, Bo Burla, Jayashree Selvalatchmanan, Jeongah Oh, Sock Hwee Tan, Mark Y. Chan, Natalie A. Mellet, Peter J. Meikle, Federico Torta, Markus R. Wenk

**Affiliations:** Singapore Lipidomics Incubator, Life Sciences Institute,* National University of Singapore, Singapore; Cardiovascular Research Institute,†† National University of Singapore, Singapore; Departments of Biochemistry,† Yong Loo Lin School of Medicine National University of Singapore, Singapore; Medicine,** Yong Loo Lin School of Medicine National University of Singapore, Singapore; National University of Singapore Graduate School for Integrative Sciences and Engineering,§ National University of Singapore, Singapore; National University Heart Centre,§§ National University Health System, Singapore; Baker Heart and Diabetes Institute,*** Melbourne, Australia

**Keywords:** lipids, mass spectrometry, liquid chromatography, quantitation, harmonization, plasma, National Institute of Standards and Technology standard reference material 1950

## Abstract

Quantitative MS of human plasma lipids is a promising technology for translation into clinical applications. Current MS-based lipidomic methods rely on either direct infusion (DI) or chromatographic lipid separation methods (including reversed phase and hydrophilic interaction LC). However, the use of lipid markers in laboratory medicine is limited by the lack of reference values, largely because of considerable differences in the concentrations measured by different laboratories worldwide. These inconsistencies can be explained by the use of different sample preparation protocols, method-specific calibration procedures, and other experimental and data-reporting parameters, even when using identical starting materials. Here, we systematically investigated the roles of some of these variables in multiple approaches to lipid analysis of plasma samples from healthy adults by considering: *1*) different sample introduction methods (separation vs. DI methods); *2*) different MS instruments; and *3*) between-laboratory differences in comparable analytical platforms. Each of these experimental variables resulted in different quantitative results, even with the inclusion of isotope-labeled internal standards for individual lipid classes. We demonstrated that appropriate normalization to commonly available reference samples (i.e., “shared references”) can largely correct for these systematic method-specific quantitative biases. Thus, to harmonize data in the field of lipidomics, in-house long-term references should be complemented by a commonly available shared reference sample, such as NIST SRM 1950, in the case of human plasma.

Lipids are a diverse class of molecules that play multiple roles in energy storage, membrane architecture, and signaling (1), and are differentially regulated in various diseases and pathological states (2). In the past few years, the lipidomic analysis of human plasma has been increasingly applied to large clinical cohorts to explore either the natural variability of the lipidome (3, 4) or the effect of disease on lipid levels (5–8).

MS-based lipidomic methods rely on either constant direct infusion (DI; “shotgun lipidomics”) or chromatographic separation [using normal phase hydrophilic interaction chromatography (HILIC), supercritical fluid chromatography (SFC), or reversed phase (RP) chromatography to separate lipids] prior to detection (9, 10). Some limited degree of separation can also be achieved in shotgun lipidomics with ion mobility (11, 12) or by using intra-source separation, i.e., favoring the ionization of selected lipid classes through solvent additives (13). MS detection is performed either “untargeted” (i.e., detection over a relatively wide *m/z* range) (14, 15), “semi-targeted” (e.g., precursor or neutral loss scanning) (16, 17), or “targeted” via selected reaction monitoring (8, 18). Lipids are then quantified by comparing against internal standards added during sample preparation (10, 19). Typically, one or two internal standards per lipid class are used (7, 15, 20, 21), as a compromise accepted by the community and due to the limited commercial availability of standards and their high cost. As a partial justification for the use of class-specific standards, ionization has been shown to depend largely on the lipid head group, especially in DI mode. However, variations in signal due to different acyl chain lengths (22), although less intense, need to be taken into consideration and can sometimes be resolved using species-specific response factors (7, 8, 23). Even a nonoptimal normalization to an internal standard belonging to a different lipid class (8) or normalization of multiple lipid classes to a single internal standard (24) can sometimes be performed using experimentally determined response factors. Stable isotope-labeled lipids are ideal internal standards (25), but their use is often limited by commercial availability and economic considerations. Fully labeled lipid extracts, e.g., from yeast grown on isotopically labeled nutrients (26), can be used as internal standards (27), but this approach is complicated by the unique lipid profiles of biological species, such as bacteria, yeast, and mammalian cells.

Previous reports have described the performances of different sample introduction methods for lipid quantitation (21), and show that HILIC, SFC, and DI methods can measure comparable phosphatidylcholine (PC) concentrations from the same sample. Different commercially available flow injection-based multiple reaction monitoring (MRM) methods similarly provide comparable PC values for concentrations (28). However, when different lipid classes and many molecular species are measured, the reproducibility of quantitative lipidomics is still limited. Begum et al. (4) previously showed that the same LC-MS method, using quasi-identical instrumental setups and methods (RP-MRM) and applied to the same sample set in two different laboratories led to comparable (albeit, not identical) results for all measured plasma lipids. Similarly, Cajka, Smilowitz, and Fiehn (14) showed that, when using the same RP method with nine different high-resolution MS systems, the actual concentrations of individual lipids could differ by up to 3-fold. Furthermore, a comparison of lipid levels determined using an untargeted RP method and a commercially available quantitative DI/ion mobility-based approach (29) found only a moderate correlation (median Pearson correlation coefficient ∼0.7); individual classes, such as cholesteryl esters (CEs), for example, were measured at vastly different concentrations.

In light of these concerns, a recent largescale inter-laboratory lipidomic study was conducted (30) to compare the lipid quantification results from a single plasma reference sample [National Institute of Standards and Technology standard reference material (NIST SRM) 1950 (31)] in various laboratories worldwide using their preferred MS-based methods. As anticipated, the study found significant disparities in the lipid concentrations reported by the participants. The lipidomics community has since come together to solve the issues related to these disparities, to improve the reporting of lipids (32, 33) and develop guidelines for lipidomic workflows (19). In particular, efforts to harmonize lipidomic workflows (34) will be supported by specific and more narrowly focused applications, such as those in human plasma lipidomics (19).

Previous proteomic (35) and metabolomic (36) studies have shown that normalization to a reference sample can substantially improve quantitative data derived from the same samples using different acquisition methods in different laboratories. Such normalization to a common reference sample was not undertaken in any of the aforementioned lipidomic studies.

Here, we report a comprehensive comparison of commonly used chromatographic and DI sample introduction approaches, coupled to high-resolution accurate MS, for the quantitative analysis of lipids in human plasma. We describe how the use of different sample introduction methods can lead to substantially different measurements of lipid levels from the same sample, even after normalization with internal standards. Finally, we demonstrate for the first time how this method-dependent quantitative bias can be overcome by normalizing to a standard reference material, thereby correcting for batch effects, different instrumental setups, and different acquisition modes (such as DI combined with high-resolution full scan vs. chromatographic separation and MRM). Our results highlight the importance and advantages of using a shared standard reference in human plasma lipidomic studies to obtain results that are comparable over time and with those from other laboratories.

## MATERIALS AND METHODS

### Chemicals

SPLASH II LIPIDOMIX Mass Spec Standard and Cer d18:1/17:0 were purchased from Avanti Polar Lipids (Alabaster, AL). The 2-propanol, acetonitrile, methanol (Optima LC-MS grade), and chloroform (analytical reagent grade) were purchased from Fisher Chemical; methyl-*tert*-butyl ether (MTBE), ammonium formate, and ammonium hydrogen carbonate (BioUltra grade) were purchased from Sigma-Aldrich; and 1-butanol (for analysis) was purchased from Merck. Deionized water was obtained from an in-house water purification system.

### Plasma samples

Two different pooled plasma samples [NIST SRM 1950, from National Institute of Standards and Technology, and pooled human plasma, K3 EDTA, here called long term reference (LTR), from Seralab, Haywards Heath, UK] were purchased and used for the experiments described. For the cross-platform analysis, we analyzed plasma samples from 21 (12 male and 9 female) healthy donors aged between 22 and 44 years. Anticoagulated blood was drawn with spray-coated K3EDTA BD Vacutainer. Plasma was collected after centrifugation and stored at −80°C.

The collection and use of all human plasma samples was approved by the Institutional Review Board of the National University of Singapore (approval numbers NUS-IRB N-17-082E and B-15-094, respectively). All study participants gave written informed consent prior to study participation and the study complied with principles of the Declaration of Helsinki.

### Sample preparation

Plasma was diluted (1+9, v/v) with 150 mM aqueous ammonium hydrogen carbonate to improve sample handling and accuracy. A variant of the MTBE-methanol-water lipid extraction method (37), optimized for plasma (15), was performed in 2 ml polypropylene Eppendorf safe-lock tubes. One hundred microliters of 1:10 diluted plasma (corresponding to 10 μl plasma) were combined with 1 ml of MTBE/methanol (7+2, v/v) containing internal standards (10 μl of SPLASH II LIPIDOMIX Mass Spec Standard and 100 pmol of Cer d18:1/17:0) and 100 μl of deionized water. Samples were agitated on an orbital shaker (Eppendorf Thermomixer C; 30 min, 1,000 rpm, 4°C), and centrifuged in an Eppendorf 5424R table-top centrifuge (14,000 *g*, 10 min, 4°C). The upper lipid-containing phase was transferred to a new safe-lock tube and evaporated in a vacuum centrifuge. The dried lipid film was reconstituted in 200 μl 1-butanol/methanol 1+1 (v/v). Both plasma samples were extracted in quintuplicates, and replicate extractions were pooled and split again to eliminate variance caused by sample preparation. Each sample was then split into 40 μl aliquots (for RP, HILIC, DI, and a backup sample), dried under vacuum, and covered with nitrogen for storage at −80°C. Four blank samples of 100 μl of deionized water were processed in the same way. Fifteen additional LTR plasma aliquots were extracted, pooled, and split for use as quality control (QC) samples throughout the analytical batches. Twenty LTR plasma aliquots were extracted without internal standards, and the resulting extracts were mixed with internal standard solution in ratios corresponding to 10, 25, 50, 75, 100, 150, and 200% of the standard sample volume (corresponding to 1–20 μl of plasma), with two replicates per point, to assess linearity of the response independent of the extraction efficiency.

For RP chromatography, samples were resuspended in 40 μl of 1-butanol-methanol (1+1, v/v); for HILIC, samples were resuspended in 40 μl of acetonitrile-water (95+5, v/v); and for DI, samples were resuspended in 500 μl of 2-propanol/methanol/chloroform (4+2+1, v/v/v) containing 7.5 mmol/l ammonium formate. See supplemental Fig. S1 for a schematic depiction of sample preparation.

### RP chromatography-MS

For RP chromatography, mobile phase A was water/acetonitrile (60/40, v/v) and mobile phase B was 2-propanol/acetonitrile (90/10, v/v), with both containing 10 mM of ammonium formate. A 1 μl sample, thermostatted to 10°C, was injected onto an Agilent Eclipse Plus C18 RP column (50 × 2.1 mm; particle size, 1.8 μm) thermostatted to 40°C in a Thermo Vanquish UHPLC system (Thermo Fisher Scientific, Bremen, Germany). Gradient elution was performed at a flow rate of 400 μl/min, beginning with 25% mobile phase B. This was increased to 60% B over 2 min, to 100% B over 10 min, and held at 100% B for 2 min. The column was then re-equilibrated for 1.8 min, giving a total run time of 15.8 min.

The LC effluent was introduced into a Thermo QExactive plus quadrupole-Orbitrap mass spectrometer via a HESI II ion source operating under the following conditions: spray voltage, 4 kV; capillary temperature, 320°C; sheath gas, 50 arbitrary units; auxiliary gas, 10 arbitrary units; spare gas, 1 arbitrary unit; probe heater, 300°C; S-lens RF level, 50. The mass spectrometer operated in full MS/ddMS^2^ (top 3) mode in positive polarity for 0 to 13 min. Automatic gain control (AGC) was set to 3E6 ions to enter the mass analyzer with a maximum injection time of 200 ms. One full scan spectrum in profile mode from *m/z* 360 to 1,000 was detected with a target resolution of 140,000 [full width at half maximum (FWHM) at *m/z* 200], followed by three sequential product ions scans of the highest abundant ions (selected from an inclusion list containing known lipid ions) at a target resolution of 17,500 (FWHM at *m/z* 200), with a normalized collision energy of 25, an isolation window of *m/z* 1.5, a fixed first mass of *m/z* 80, and an AGC target of 1E3. Ions with charge other than +1 were excluded, and isotopes were excluded. The dynamic exclusion time was set to 2 s. A common polysiloxane background ion (*m/z* 536.16536) was used as lock mass in full scan mode.

### HILIC-MS

HILIC, similar to (38), was performed on a Waters BEH HILIC (100 × 2.1 mm; particle size, 1.7 μm), thermostatted to 40°C in a Thermo Vanquish UHPLC system. Mobile phase A was 40 mM aqueous ammonium formate, adjusted to pH 4 with formic acid, and mobile phase B was acetonitrile. Five microliters of sample, thermostatted to 10°C, was injected onto the column, and gradient elution started at 96% mobile phase B for 1 min, changing to 70% B over 6 min, and back to starting conditions over 0.1 min. The column was then re-equilibrated for 2 min. The LC effluent was introduced into a Thermo QExactive plus quadrupole-Orbitrap mass spectrometer via a HESI II ion source operating under the following conditions: spray voltage, 3.8 kV; capillary temperature, 350°C; sheath gas, 50 arbitrary units; auxiliary gas, 10 arbitrary units; spare gas, 0 arbitrary units, probe heater, 330°C; S-lens RF level, 50. The mass spectrometer operated in full MS/ddMS^2^ (top 8) mode in positive polarity. AGC was set to 1E6 ions to enter the mass analyzer with a maximum ion time of 200 ms. A full scan spectrum in profile mode from *m/z* 360 to 1,000 was detected with a target resolution of 70,000 (FWHM at *m/z* 200), followed by eight centroid product ion scans of the highest abundant ions (selected from an inclusion list containing known lipid ions) at a target resolution of 17,500 (FWHM at *m/z* 200), with a normalized collision energy of 20, an isolation window of *m/z* 1.5, a fixed first mass of *m/z* 80, and an AGC target of 8E3. Ions with a charge other than +1 were excluded, isotopes were excluded, and dynamic exclusion time was set to 2.5 s. A common polysiloxane background ion was used as lock mass (*m/z* 536.16536) in full scan mode.

### DI-MS

A DI chip-based nano-electrospray device (TriVersa NanoMate; Advion Biosciences, Ithaca, NY), controlled by Chipsoft 8.3.1 software, was interfaced to a Thermo QExactive plus quadrupole-Orbitrap mass spectrometer, similar to that described previously (15, 20). Ten microliters of sample, thermostatted to 4°C in a 96-well plate, were infused via 4.1 μm spraying nozzles for 2.5 min. Backpressure was set to 1.25 psi and ionization voltage to 1.25 kV. After 30 s of spray stabilization, FT-MS spectra in the range of *m/z* 400 to 1,000 were acquired for 1 min in profile mode at a resolution setting of 140,000 (FWHM at *m/z* 200) with an AGC setting of 1E6 and maximum ion time of 200 ms. For the next 1 min, product ion scans in centroid mode were acquired in data-independent analysis mode, using a precursor list starting at *m/z* 400.25, with increments of *m/z* 1.001, until *m/z* 999.849, with a resolution setting of 17,500 (FWHM at *m/z* 200), an AGC setting of 1E5, a normalized collision energy of 20, an isolation window of *m/z* 1, and a fixed first mass of *m/z* 80. Samples were analyzed first in positive, then negative, ion mode, using lock masses of common background ions (*m/z* 536.16536 and *m/z* 680.48022 in positive ion mode, and *m/z* 529.46262 in negative ion mode) in full scan mode.

### Lipid identification and quantification

The lipid nomenclature used here is based on the LIPID MAPS classification system (1, 39), using shorthand nomenclature adapted for mass spectrometric analysis (40), and lipids are reported at the bond type level.

For RP analysis, lipids were identified using Lipid Data Analyzer 2 (41, 42), relying on accurate mass, tandem mass spectrometric behavior, and retention time. HILIC data were processed with the proprietary vendor software (Thermo XCalibur 3.0.63), identifying lipids based on class-specific retention time and accurate mass within a 20 ppm window (to ensure that the entire profile peak was integrated and that all isotopic interferences were included in the area value). M+2 isotope correction was performed in R (version 3.6.0) using a custom-made R script (https://github.com/SLINGhub/Manuscript_Triebl_2019) and the package “envipat” (version 2.4) (43). DI data were processed by LipidXplorer 1.2.7 (44), identifying lipids by their accurate mass and tandem mass spectra (tolerance 5 ppm for MS^1^ and 20 ppm for MS^2^). The output was manually filtered to exclude highly improbable lipids identified with none of the other methods and duplicate identifications (structural isomers with different fatty acyl compositions).

Concentrations of lipids were obtained by normalizing the de-isotoped lipid peak areas (for RP and HILIC) or peak intensities (for DI) to those of the class-specific internal standard. Plasmenyl species were normalized to the respective diacyl internal standard. No additional species-specific response factor correction was used [corresponding to Level 2 of the guidelines set forth by the Lipidomics Standards Initiative (45)].

### QC

We followed guidelines outlined previously (19, 46) for analytical quality assurance and QC. The sample run order is shown in supplemental Table S4. Reproducibility was evaluated by 10 replicate injections of a representative plasma extract spaced throughout each analytical batch. Four replicate blank extractions were used to identify common contaminations. A dilution of lipid extract relative to internal standard solution was used to investigate concentration-response behavior. For every method, we quantified only those lipids that could be reproducibly detected [coefficient of variation (CoV) <20%] that were not present in the blank extracts (<10% of QC), and that showed sufficient response to dilution (*R*^2^ > 0.9 in the range of 50–200% of sample volume used, i.e., 5–10 μl).

### Statistical analysis

**Fig. 1.**
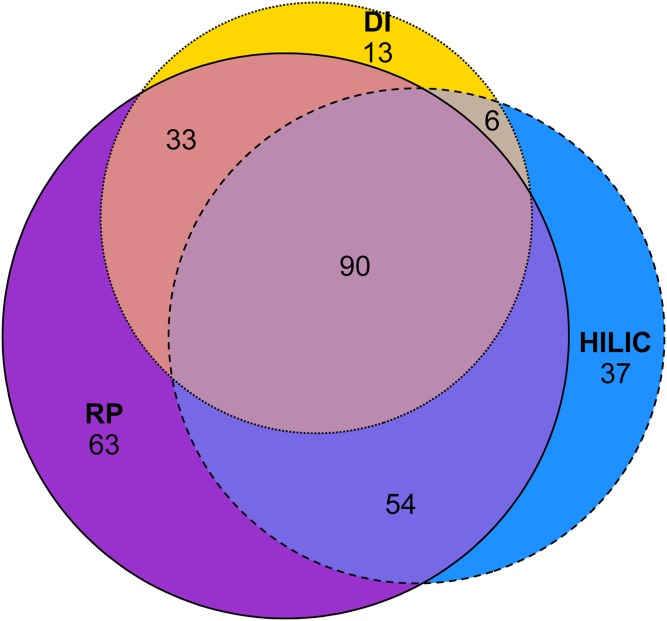
Number of lipids quantified with RP, HILIC, and DI.

Principal component analysis (PCA) plots were created online using MetaboAnalyst (www.metaboanalyst.ca) (47–49) using the following parameters: sample normalization, none; data transformation, none; data scaling, none. Data in supplemental Table S1 were created using MetaboAnalyst’s volcano plot function, with the following parameters: fold change threshold, 1.1; *P*-value threshold, 0.05, FDR-adjusted; group variance, equal. The Euler diagram in Fig. 1 was created online using eulerr (50). All other figures were created in Microsoft Office 2016.

### Normalization to standard reference sample

For every lipid measured with each of the three methods, the calculated concentrations of the LTR plasma sample $\left(c{\;}_{\mathrm{Sample}\;j}^{\;\mathrm{Analyte}\;i}\right)$were normalized to the calculated concentrations of the NIST SRM 1950 measured with the same method $\left({\bar{c}}_{\mathrm{NIST}}^{\;\mathrm{Analyte}\;i}\right)$, using consensus values [median of means (MEDM)] for NIST SRM 1950 $\left[c{\left(\mathrm{Ref}\;\mathrm{NIST}\right)}_{}^{\mathrm{Analyte}\;i}\right]$ to obtain final absolute concentrations (30):

$$c_{\;\mathrm{Sample}\;j}^{\;\mathrm{Analyte}\;i}\left(\text{normalized}\right)=\frac{c{\;}_{\mathrm{Sample}\;j}^{\;\mathrm{Analyte}\;i}}{\bar{c}{\;}_{\mathrm{NIST}\;}^{\;\mathrm{Analyte}\;i}}\times c{\left(\mathrm{Ref}\;\mathrm{NIST}\right)}^{\mathrm{Analyte}\;i}$$

Comparability with other studies is already given by expressing the concentration relative to a reference sample. Multiplication with the consensus value is an optional step, which creates absolute values, such as those in supplemental Fig. S2, rather than dimensionless relative quantities. We would like to clarify that using a multiplier is not required for normalization by an external reference standard because the purpose of it is to express experimental measures relative to a reference material. Although the current consensus values for lipids in NIST SRM 1950 are derived from individual measurements with very high variability (30), they will in the future most likely be improved by more concordant and reliable values that will be used as a multiplier in this equation. In such a case, this will allow a retrospective comparison of data obtained in previous studies, e.g., using tools such as “LipidQC,” which compares results generated with published consensus values (51).

### Interlaboratory meta-analysis and cross-platform analysis

Data from a previously published natural variation study (4), where the same sample set (478 human plasma samples from healthy individuals) was analyzed with the same RP-MRM method in two different laboratories, were used to determine whether a common reference sample can correct for quantitative differences. A pooled QC sample (prepared by mixing aliquots of all the study samples and measured in both sites) was used to align the results.

Additionally, the applicability of normalization for cross-platform comparability was investigated by measuring 22 human plasma samples, extracted with internal standards, as described elsewhere (52), together with a commercially available reference sample (NIST SRM 1950) with both the DI high-resolution MS (DI-HRMS) method described above and an RP-MRM method (53).

For both comparability studies, lipid concentrations were calculated independently for sites/methods, and median fold correction was performed to normalize the study samples to the common reference sample (either the pooled QC sample or NIST SRM 1950). PCA and correlation plots were created as described above.

### Data availability

The MS raw data, properties, mass lists, and fragmentation rules for Lipid Data Analyzer; the Xcalibur processing method and the R-based data extraction and isotope correction script for HILIC data as well as the import settings and mfql files for Lipid­Xplorer are available from Zenodo (doi: 10.5281/zenodo.3346646; https://zenodo.org/record/3346646). R scripts used for processing the HILIC-HRMS dataset are also available (https://github.com/SLINGhub/Manuscript_Triebl_2019).

## RESULTS

### Lipidomic coverage when using different sample introduction methods

Analytical quality assurance and QC are paramount to ensure the integrity of lipidomic results. We base our quantitative results on data filtering for each lipid, which consists of *1*) a reproducible measurement, *2*) an absence of signal in the extracted blanks, and *3*) a linear response to dilution. Typically, the number of detectable *m/z* values corresponding to lipid ions is far greater than the number of identified lipids that pass all QC checks (e.g., 450 detected vs. 142 quantifiable lipids in the case of the DI dataset).

From our results, different sample introduction methods (RP, HILIC, DI) provided different lipid class and species coverage in plasma, as shown in **Fig. 1**. Most lipid classes were detectable with all three methods when using the positive ion full-scan mode on a high-resolution mass spectrometer. Some classes were not detectable when using a specific method, such as ceramides with DI or DAG and CEs with HILIC; the latter might be explained by the fact that these classes are not retained in HILIC but elute very early in regions with high ion suppression, caused by charge competition with highly abundant TAG.

The highest number of lipids (240 glycerolipids, glycerophospholipids, and sphingolipids) was detected with RP, which is not unexpected, as chromatographic separation generally improves the detectability of low-abundance lipids and lipids with low ionization efficiency. This is particularly noticeable for pairs of saturated and monounsaturated lipids, where saturated species are in lower abundance (e.g., SM d34:0, PC 34:0) as compared with their monounsaturated analogs (SM d34:1, PC 34:1). Saturated species can often only be quantified in RP, when they are chromatographically separated from their unsaturated analogs. Comparatively, in HILIC and DI, the signal intensity of saturated species is mainly contributed by the isotopic interference from unsaturated analogs, and the saturated species are then often removed by isotope correction algorithms. Our DI approach provides the lowest lipid coverage [n = 142; comparable with other published methods (20) when filtered by reproducibility], as signals from low-abundant lipids are often not reproducible in replicate measurements nor do they respond linearly to dilution.

We compared the results obtained in positive-ion mode with those in negative-ion mode [often preferred for phospholipid analysis (15, 20)] and found that positive ion mode leads to an overall higher lipid coverage. Although negative-ion mode often performs better for phospholipids, such as PE, LPE, or PI, other lipid classes, such as TAG, DAG, or CE, do not ionize at all or only very poorly in negative-ion mode. Acquiring a sample in both polarities can provide both high coverage and selectivity. However, for the best comparison of our experimental data, further analyses were conducted using only the results from positive-ion mode.

### Characteristics of different sample introduction methods

**Fig. 2.**
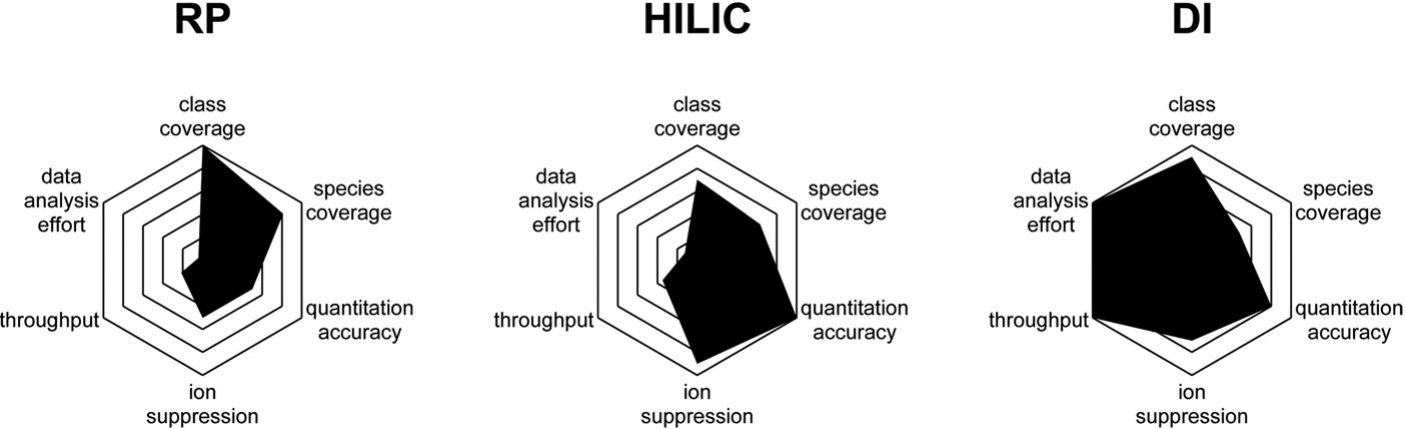
Semi-quantitative comparison of sample introduction methods in lipidomics shows the strengths and weaknesses of each method. Whereas RP affords the highest lipidomic coverage, HILIC offers the highest quantitation accuracy due to the co-ionization of analytes with internal standards without interference from other lipid classes (which are chromatographically separated). Analytical and data analysis throughput is best with DI.

**Figure 2** shows a qualitative comparison of the three sample introduction methods. Whereas RP provides the highest number of quantifiable lipid classes and lipid species, it is also the most time- and labor-intensive of the three methods. Even the fastest RP methods, which still provide sufficient chromatographic resolution to be efficiently used in complex matrices like plasma, have injection intervals of more than 10 min (4, 7, 8) and have a time-consuming data analysis procedure. Chromatographic separation in RP is driven mainly by hydrophobicity; thus, many molecular species of one class typically elute over a large retention time, and there often is no coelution with internal standards. The biggest advantage of RP is its very high chromatographic resolution, with the capability to even resolve structurally similar molecules (e.g., isomeric plasmalogen- and alkenyl-phospholipids) (8); this is not possible with HILIC or DI. The advantage of HILIC (and SFC, where the principles of separation are similar) is that chromatographic separation is determined primarily by the lipid head group, with there being very little to no separation within molecular species of one class. This means that different lipid classes are chromatographically separated, reducing inter-class ion suppression effects, while all lipids of one class, including the respective internal standards, elute and ionize at the same time; this is an important prerequisite when quantitation is the aim (10). HILIC data, as well as DI data, must also be mathematically processed to correct for the naturally occurring isotopologues (25), unless ultra-high mass resolution (>>100,000 FWHM) is used (54).

DI (or “shotgun”) omits chromatography altogether and is based on a constant infusion of the sample directly into the mass spectrometer. The use of a nano-electrospray source can additionally improve ionization efficiency and increase detectability and sensitivity, as compared with normal flow ESI (55), while keeping sample consumption minimal. DI provides the shortest runtimes, as, in our approach, injection intervals were kept under 3 min. Data analysis is highly automated, and large batches can be processed much faster and more reliably than with chromatography-based methods; this is due to the lack of chromatographic peaks and issues related to their correct and reproducible integration. However, for the same reason, lipid identification relies solely on accurate mass and is more prone to interference. Whereas tandem mass spectra can be additionally used for confirmation, these are often complicated by cofragmentation of other (lipid) ions within the isolation window. Our results show that DI reliably quantifies the lowest number of lipids (n = 142), likely due to ion suppression and charge competition effects from high-abundance compounds, which are less pronounced in chromatography-based methods.

### Different introduction methods can influence lipid quantitation

The two samples initially used in this study are commercially available pooled plasma from 50 and 100 healthy donors, respectively. These samples show notable differences in their lipid profiles, presumably attributed to differences in blood collection (EDTA vs. lithium-heparin anticoagulants), the donor population (ethnicity and health status), and sample processing. These differences are nevertheless detectable at the lipid-species level using unsupervised statistical methods with all three sample introduction methods (supplemental Table S1).

**Fig. 3.**
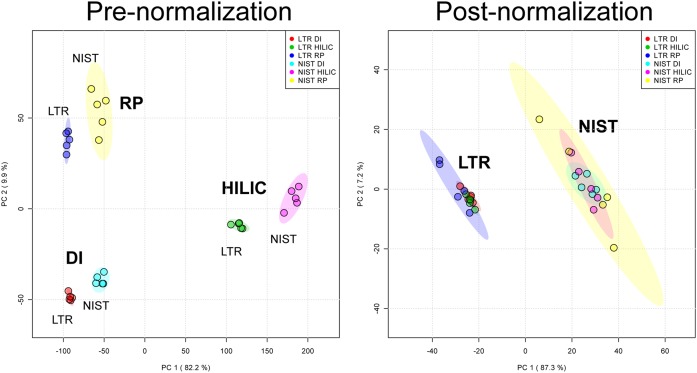
PCA score plots of lipid concentrations measured for two plasma samples using three different methods (RP, HILIC, and DI) before (left) and after (right) normalization to a reference sample. Plots are based on the concentrations of 75 lipids identified with each method.

When measuring the same lipid extract with the three different sample introduction methods, using the same mass spectrometer and the same internal standards mixture, the final calculated concentrations substantially differ between the methods. **Figure 3** (left panel) shows a plot of the PCA scores of the two different lipid extracts, measured in quintuplicate with the three different methods. Only lipids that could be reliably quantified with each method and for which NIST consensus values (30) are available (n = 75) were considered for this analysis. While the two different plasma samples can be discriminated with any method, the same sample measured with different methods can also be clearly discriminated using unsupervised statistical methods.

The differences in the estimated final concentrations were more pronounced for some lipid classes than others (supplemental Figs. S2–S4). For example, many lysophosphatidylcholine (LPC) and PC species showed good agreement in the concentrations obtained with all three methods, as previously shown (21). This could be attributed to their relatively high concentrations in plasma and their high ionization efficiency as compared with other lipid classes.

We next calculated the overall CoV across 15 measurements (five replicates for each method) as a measure of variability between the lipid concentrations obtained with different methods (supplemental Fig. S3). Whereas most lipid species showed large variability (CoV, 30–80%), the overall variability for the three endogenous lipid species that were normalized to the corresponding stable isotope-labeled analog (LPC 18:1, SM d36:2, and TG 48:1) was below 12%, indicating similarity in their calculated concentration values across the three different methods. Our results thus confirm that the use of species-specific (but not class-specific) standards can efficiently correct for ionization differences. This effect is only noticeable for the corresponding nonlabeled species and not for other coeluting species of the same class. However, the use of stable isotope-labeled internal standards is limited by their availability (synthetic individual lipid species or fully labeled lipid extracts) and by the cost associated with routinely using multiple stable isotope-labeled lipids per class.

### Normalization to a reference sample removes method-dependent quantitation differences

**Fig. 4.**
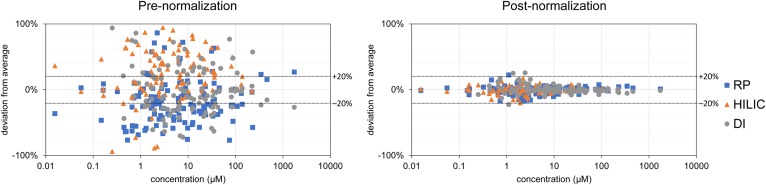
Normalization to standard reference sample effectively removes method-dependent quantitative bias, represented as differences from the average measured concentration. Normalization is independent of lipid concentration. Left side shows large differences in lipid concentrations measured by different sample introduction methods, which are removed after normalizing to a common reference sample. See supplemental Fig. S4 for species-specific depiction. Only lipids detectable with more than one sample introduction method are shown.

We investigated whether normalization to a commonly measured reference material could correct for method-dependent systematic differences in the reported lipid levels. A reference material is a homogeneous pool of unprocessed matrix that is representative of the study matrix (in this case, plasma). It is usually analyzed at regular intervals among the study samples, predominantly as a QC measure. The reference material must be prepared in the same way as the study samples in each analytical batch, starting from the preparation of aliquots and lipid extraction. The NIST SRM 1950 (31) was chosen as a reference material for our study because of its wide availability. For each method previously described, lipid concentrations were then normalized by dividing the measured concentration of each lipid in the study sample by the measured concentration in the NIST SRM 1950 sample, and (as an optional step) multiplying this number by the reported consensus concentration to create absolute values. A similar process has been recently reported for proteomic measurements (35). We show that, after this normalization, unsupervised statistical methods can no longer clearly differentiate if the same plasma sample is measured with different sample introduction techniques (**Fig. 3**, right panel), with differences in the concentrations significantly minimized (**Fig. 4**, supplemental Fig. S5). The overall CoV (calculated across methods for all the lipid species) approaches the technical variability (supplemental Fig. S3). Furthermore, the effectiveness of this normalization is not dependent on lipid abundance, as shown in Fig. 4.

Supplemental Table S1 shows that, after normalization, all three methods find comparable differences between the two different plasma samples.

**Fig. 5.**
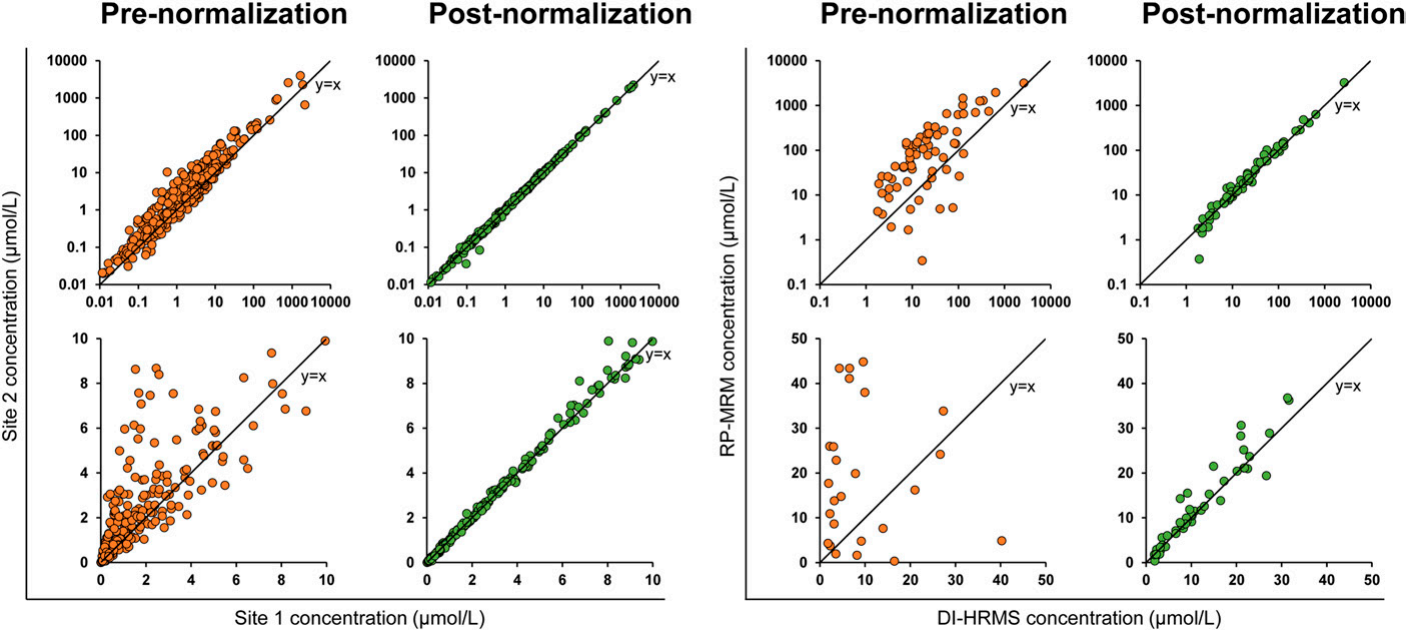
Inter-site and inter-method variability is improved after normalizing to a common reference sample. Comparison of lipid concentrations measured in the same samples with the same RP-MRM method in two different laboratories (Site 1 and Site 2, respectively; left), and with a DI-HRMS method and a RP-MRM method (right). Top row shows log-scaled axes. Bottom row shows a zoomed inset on a linear scale for lipids present at low concentrations. Black line indicates perfect comparability (y = x). Normalization to a common reference sample harmonizes results at different sites using the same method (left). Some quantitative bias remains after normalization when the samples are analyzed with different instruments and methods (right).

We then applied the same approach to understand how comparable the quantitative lipid profiles of human plasma samples from healthy individuals (n = 22) are, when measured with two extremely different MS platforms (RP-MRM and DI-HRMS) after correcting with a standard reference sample. For this kind of analysis, at least one reference standard sample should be included in each analytical batch together with the study samples (supplemental Table S5). Initially, the concentrations measured by the two different approaches were poorly comparable (**Fig. 5**). However, as anticipated, normalization using the standard reference (NIST SRM 1950) greatly improved the agreement in the concentration values (Fig. 5; supplemental Figs. S6, S7); albeit, there remains some quantitative bias, possibly due to signal interferences in one of the methods used and affecting a small number of species.

We then investigated the use of standard reference samples to normalize data from large human cohorts. We reanalyzed the data obtained from an earlier study (4), where the same cohort (plasma samples from 478 healthy people) was analyzed by two different laboratories with the same RP-MRM method on two different QqQ-MS models. Instead of using the NIST SRM 1950, an internally pooled plasma sample (Pooled QC in supplemental Table S5) was measured in both laboratories in each analytical batch and then used as reference standard to align the lipid concentration values using a median fold transformation. We found that normalization greatly improved the correlation between the lipid levels (Fig. 5), and effectively removed the quantitative bias (see also supplemental Figs. S6, S7), allowing comparability between the two sets of results obtained in different laboratories.

## DISCUSSION

The lipidomics field continues to suffer from limitations associated with reproducibility and comparability of quantitative data, particularly across different institutions (30). However, researchers in the field are directing their efforts toward understanding the sources of such analytical biases and establishing a more accurate quantitative lipidomic approach. This will be essential for the translation of MS-based lipidomics into clinical research and laboratory medicine.

Here, we have compared the advantages and disadvantages of commonly used chromatographic (RP and HILIC) and DI sample introduction methods for the quantitative analysis of lipids in human plasma using the same reference materials. We show that different methods generate different quantitative values, even when the same sample extracts are analyzed with appropriate class-specific internal standards. In line with previous studies, this result calls into question the direct comparability of lipidomic data generated by different methods (29, 30). Even though our results were obtained from aliquots of the same lipid extract, thereby limiting the measured variability only to methodological differences, additional quantitative differences are introduced by the use of different internal standards, sample preparation procedures, chromatographic separation, and MS detection approaches. Furthermore, even when we maintained very similar MS detection parameters (e.g., high-resolution full scan in positive polarity), we still saw variances in our results. Other commonly used lipidomic methods, based for example on t-SIM or MRM, rely on different detection principles, likely introducing additional variability to quantitative results. There is currently no accepted “gold standard” for MS-based lipidomics, and the choices involved with each analytical method (chromatographic or DI, instrument type, full scan or MRM, type and number of internal standards, and the use of species-specific response factors) rely to a great extent on personal preference and laboratory experience and on the specific advantages and disadvantages of each method for a specific study (e.g., lipid coverage for RP, coelution with reduced ion suppression for HILIC, or samples high throughput for DI; Fig. 2).

Even when taking into account the inherent limitation of quantitative lipidomics due to the limited availability of labeled internal standards, our results suggest that the large disparities in plasma lipid concentrations reported by different laboratories are primarily caused by the use of different analytical methods. Based on our results, CEs seem to be greatly affected by ion suppression and charge competition effects, with calculated concentrations differing by more than 50% for high-abundance species when comparing DI and RP results, whereas these species are undetectable in HILIC. By normalizing CE levels using a standard reference sample measured with both methods, this difference was reduced to less than 5%. Even for TAG, the higher concentration values measured in HILIC (compared with RP and DI and presumably caused by complex charge competition effects at the beginning of the HILIC gradient) are reduced to within 10% of average concentrations after normalization with the reference sample. These results suggest that differences in measured concentrations are strongly dependent on the analytical method and, because these differences are consistently observed across different samples, we can apply a normalization using a reference sample as a correction.

The molecular species belonging to DAG, TAG, and CE lipid classes exhibit large differences in ionization, which are highly dependent on carbon chain length and degree of unsaturation (7, 8, 23, 56). Therefore, studies have suggested the use of response factor correction as an alternative to standard references to improve quantification. However, correctly identifying a particular response factor for each sample is a more cumbersome approach and requires extensive experiments with synthetic standards, additional injections, and further calculations. Additionally, it is only applicable to a single method (minor changes in solvent composition or instrumental conditions might alter response). However, we show that these discordances can be corrected by normalization to a reference material. The obtained normalized values will be relative to the reference standard sample, and can be converted to absolute concentration units once values for the reference material are available (19). We therefore propose that lipidomic experiments should be designed to include an appropriate reference material, such as a laboratory-specific LTR or commercially available standards (e.g., NIST SRM 1950), to avoid method-specific quantitative biases and improve data comparability. The utility of this approach will be especially relevant for longitudinal studies (when samples are collected and analyzed over long periods of time), for multicenter validation studies, and for projects involving the use of several instrumental setups. Even if this correction will help in harmonizing results obtained in different conditions, the operators will have to be careful when selecting peaks during the data analysis step and make sure that there is a perfect correspondence between the study samples and the reference sample species used for normalization.

### Limitations of the study and recommendations

The normalization-based approach proposed here is possible only for compounds found in both study samples and the reference materials. This is the reason why the standard reference should be representative of the same matrix of the study samples; in fact, it should be as similar as possible. In the case of plasma/serum lipidomics, it should not be too difficult to satisfy this requirement. However, we foresee possible situations where some lipid species may be present in the sample studies at a certain level but not at all, or at vastly different concentrations, in the external reference standard (due to regulation of a specific pathway and/or to a disease/mutation, etc.). If these lipids are relevant to the study and need to be quantified, a different reference standard should be considered and included. In this case, a pool across aliquots from all the study samples would be more appropriate for normalization. This reference standard (pooled QC sample) is also routinely used to correct for batch effects and to estimate the quality of the analytical procedure. It represents a QC sample with the average abundance of the lipid species present in all the samples. In order to compare results from multiple studies (as shown here for the multicenter or multi-platform comparisons), it is essential that the pooled QC sample is shared between the participating laboratories or instruments. Another challenging situation might arise when the matrix of the study and the reference standard are different (e.g., liver extracts in study samples and NIST SRM 1950 plasma as a reference). In that case we suggest to use a pooled QC generated by the study samples to recalibrate the results. If an aliquot of these pooled QC samples is stored until proper matrix-specific reference materials are available and characterized (NIST is currently generating new standard reference samples for tissues other than plasma), future recalibration will always be possible. The main goal of our approach is to express experimental measures relative to a common reference to increase overall comparability. It should be noted that normalization to concentration consensus values previously published (30) is not a requirement for this method and it should only be used as a “temporary remedy” while more accurate values are being generated by ongoing initiatives.

We are aware that limitations of using the NIST SRM 1950 might be also linked to its cost and to the fact that its extensive use might one day limit its availability. As an alternative solution, a cheaper laboratory-specific LTR or, as shown in our multicenter comparison, an internally generated reference sample (pooled QC) can be considered. These are alternatives that can correct for analytical differences when the NIST standard is not available. In the case of human plasma lipidomics (19), the NIST SRM 1950 is, however, an immediately available option to harmonize measurements on a worldwide scale, because: *1*) it is pooled human plasma; and *2*) in principle, it is accessible to all the laboratories. In order to reduce NIST SRM 1950 consumption (and therefore cost to individual laboratories) one could propose a viable compromise: measure the ratio between the same lipid species in the pooled QC (or laboratory-specific LTR) and in the NIST SRM 1950 once, in order to recalibrate to the NIST reference all the measurements previously normalized to its in-house alternative. In this case, the experimental samples are calibrated to their respective internally generated (cheaper) reference materials, which are the converter to the NIST (master) reference.

We demonstrate that species-specific standards are ideal and therefore extremely valuable when they are available. More complex mixtures of labeled lipids are therefore another plausible alternative for reference materials. However, such mixtures would also have certain limitations, for example, variations depending on the production batch, stability, and careful optimization of the system in use for lipidomic measurements. Multiple internal standards per lipid class is an attractive way forward to bridge the gap between class- and species-specific internal standard. It will need careful validation to circumvent difficult decisions (e.g., to which of the different standards in one class should an endogenous species be normalized?). The choice between different alternatives would again affect the reproducibility between laboratories. There is no perfect solution yet, so we would suggest use of the reference sample described here together with multiple internal standards, when they are available.

Reporting lipidomic results relative to a commonly available worldwide reference sample (such as the NIST SRM 1950) requires limited additional costs, time, and work, especially when used for the analysis of large clinical cohorts. However, this approach has enormous benefits for results to remain comparable over time. Importantly, so long as the reference materials are measured with the study samples, these data normalizations and comparisons can be performed at a later stage, when reference materials will be characterized in more detail. Furthermore, when more reliable consensus values are available for these reference samples, calculations can be made to obtain absolute concentrations across all studies that employed the same reference sample. The approach shown should be widely applicable, not only to plasma lipidomics, but to other studies of endogenous analytes with limited availability of identical stable isotope standards.

## Supplementary Material

Supplemental Data
