# Supplementary material for: Shared reference materials harmonize lipidomics across MS-based detection platforms and laboratories
Source: J Lipid Res. 2019 Nov 15;61(1):105–15. doi: 10.1194/jlr.D119000393 (PMC6939597; doi:10.1194/jlr.D119000393)
Supplement: Supplemental Data [file supp_61_1_105__index.html]

Shared reference materials harmonize lipidomics across MS-based detection platforms and laboratories — Cross-platform and inter-lab harmonization of lipidomics — Shared reference materials harmonize lipidomics across MS-based detection platforms and laboratories — Supplemental Data 

# Shared reference materials harmonize lipidomics across MS-based detection platforms and laboratories

## Supplemental Data

- Supplemental Data - contains information on data availability, 5 supplemental table, and 7 supplemental figures
