# Supplementary material for: Shared reference materials harmonize lipidomics across MS-based detection platforms and laboratories
Source: J Lipid Res. 2019 Nov 15;61(1):105–15. doi: 10.1194/jlr.D119000393 (PMC6939597; doi:10.1194/jlr.D119000393)
Supplement: Supplemental Data [file supp_D119000393_155837_2_supp_428532_q0xl8q.pdf]

SHARED REFERENCE MATERIALS HARMONIZE LIPIDOMICS ACROSS MS-BASED DETECTION PLATFORMS AND LABORATORIES

Alexander Triebl[1,2], Bo Burla[1], Jayashree Selvalatchmanan[1,2], Jeongah Oh[1,3], Sock Hwee Tan[4,5], Mark Y. Chan[2,4,5,6], Natalie A. Mellet[7], Peter J. Meikle[7], Federico Torta[1,2]*, Markus R. Wenk[1,2]*

Author Affiliations

[1] Singapore Lipidomics Incubator, Center for Life Sciences, National University of Singapore; 28 Medical Drive, Singapore 117456, Singapore

[2] Department of Biochemistry, Yong Loo Lin School of Medicine, National University of Singapore; 8 Medical Drive, Singapore 117596, Singapore

[3] NUS Graduate School for Integrative Sciences and Engineering, 21 Lower Kent Ridge, Singapore 119077 Singapore

[4] Department of Medicine, Yong Loo Lin School of Medicine, National University Hospital, Singapore

[5] Cardiovascular Research Institute, National University of Singapore, Singapore

[6] National University Heart Centre, National University Health System, Singapore

[7] Baker Heart and Diabetes Institute, 75 Commercial Road, Melbourne, VIC 3004, Australia

Corresponding Authors:

Federico Torta, Department of Biochemistry, Yong Loo Lin School of Medicine, National University of Singapore, Center for Life Sciences, 28 Medical Drive, Singapore 117456, Singapore. Tel: +6565168174, Email: bchfdtt@nus.edu.sg

Markus R Wenk, Department of Biochemistry, Yong Loo Lin School of Medicine, National University of Singapore, Center for Life Sciences, 28 Medical Drive, Singapore 117456, Singapore. Tel: +6565168174, Email: bchmrw@nus.edu.sg

DATA AVAILABILITY

The following data associated with this manuscript is available from zenodo:

https://doi.org/10.5281/zenodo.3346646 / https://zenodo.org/record/3346646

1. **Raw data**
   1.1. RP data
   1.2. HILIC data
   1.3. DI data
2. **Data processing**
   2.1. RP data processing
      2.1.1. Lipid Data Analyzer properties
      2.1.2. Lipid Data Analyzer mass list
      2.1.3. Lipid Data Analyzer fragmentation rules
   2.2. HILIC data processing
      2.2.1. XCalibur processing method
      2.2.2. Data extraction from Xcalibur and isotope correction
   2.3. DI data processing
      2.3.1. LipidXplorer import settings
      2.3.2. LipidXplorer mfql files positive
      2.3.3. LipidXplorer mfql files negative
3. **Results**
   3.1. RP_HILIC_DI_results
   3.2. Inter-site analysis
   3.3. Cross-platform analysis

SUPPLEMENTAL TABLES

Supplemental Table S1: Significantly up-/downregulated lipids in LTR plasma compared to NIST plasma sample, normalized values, as measured with RP, HILIC, and DI. FC, fold change, p-value FDR-adjusted, n.s., p>0.05.

	RP		HILIC		DI	
	**FC**	**p-value**	**FC**	**p-value**	**FC**	**p-value**
**LPC 15:0**	1.20	8.7E-04	1.18	3.3E-03	1.42	2.2E-06
**LPC 16:0**	1.36	7.9E-08	1.34	1.9E-08	1.31	1.7E-08
**LPC 16:1**	0.76	6.2E-03	0.67	1.7E-08	0.65	8.6E-08
**LPC 18:0**	1.77	1.0E-07	1.68	1.6E-10	1.61	7.7E-09
**LPC 20:3**	1.25	2.6E-04	1.35	3.8E-07	1.51	1.5E-05
**LPC 22:6**	1.61	1.4E-04	1.50	1.3E-05	2.22	1.5E-05
**PC 30:0**	0.82	5.4E-04	0.84	1.2E-03	0.79	1.2E-05
**PC 32:1**	0.61	1.3E-05	0.61	1.0E-07	0.62	4.1E-11
**PC 34:1**	0.88	4.1E-03	0.90	2.2E-04	0.87	1.6E-08
**PC 36:1**	0.89	8.3E-03	n.s.		0.82	8.5E-07
**PC 36:3**	0.80	3.5E-04	0.82	1.1E-04	0.81	8.6E-08
**PC 36:4**	0.91	2.8E-02	n.s.		n.s.	
**PC 36:5**	0.79	6.4E-05	0.89	5.8E-03	n.s.	
**PC 38:5**	0.74	1.6E-05	0.79	1.3E-05	0.85	2.2E-05
**PC 38:6**	0.87	2.3E-02	0.86	7.4E-05	0.89	4.8E-05
**PC 40:7**	0.58	1.4E-03	0.63	2.5E-06	0.88	3.3E-03
**PC 40:8**	n.s.		0.83	1.0E-02	n.s.	
**PC O-32:1/31:1**	n.s.		n.s.		1.23	6.4E-03
**PC O-34:2/33:2**	n.s.		1.11	4.1E-03	n.s.	
**PC O-34:3/33:3**	1.24	1.1E-03	1.22	2.0E-03	1.35	3.7E-07
**PC P-35:2/34:3**	0.75	2.1E-03	0.79	3.8E-07	0.78	4.9E-07
**PC O-36:1/35:1**	n.s.		n.s.		0.85	2.7E-03
**PC O-36:3/35:3**	0.83	2.9E-03	n.s.		0.77	6.4E-03
**PC O-36:5/35:5**	n.s.		1.11	3.4E-02	1.22	1.9E-06
**PC O-38:5/37:5**	n.s.		0.88	8.5E-04	0.89	1.2E-06
**PE 36:2**	n.s.		n.s.		0.84	3.7E-05
**PE O-36:5**	1.26	2.4E-05	1.31	1.9E-05	1.28	3.8E-02
**PE O-38:5**	1.18	4.0E-04	1.17	6.0E-04	n.s.	
**PE O-38:6**	n.s.		1.12	3.6E-03	n.s.	

	RP		HILIC		DI	
	**FC**	**p-value**	**FC**	**p-value**	**FC**	**p-value**
**PE O-38:7**	1.10	9.4E-03	1.26	3.2E-05	n.s.	
**SM d33:1**	1.10	1.8E-02	n.s.		n.s.	
**SM d38:2**	1.16	4.3E-04	1.11	1.9E-05	1.13	1.6E-04
**SM d40:3**	0.84	1.7E-03	0.82	6.4E-07	0.72	1.9E-04
**SM d42:3**	1.15	1.9E-03	n.s.		n.s.	
**TAG 46:2**	0.80	5.6E-06	0.68	1.3E-04	0.81	2.8E-03
**TAG 48:1**	0.74	7.9E-08	0.74	1.0E-06	0.75	5.9E-06
**TAG 48:2**	0.72	3.0E-06	0.69	3.8E-07	0.72	6.3E-06
**TAG 50:1**	0.85	2.0E-04	0.89	2.6E-05	0.86	1.7E-05
**TAG 50:2**	0.79	4.6E-07	0.79	3.8E-07	0.78	4.6E-08
**TAG 50:3**	0.75	3.2E-07	0.74	2.9E-06	0.74	8.5E-08
**TAG 50:4**	0.74	5.8E-06	0.74	1.9E-05	0.79	1.5E-06
**TAG 50:5**	0.76	7.5E-05	0.78	2.6E-04	0.78	2.7E-06
**TAG 51:2**	0.79	1.8E-07	0.76	1.6E-05	0.76	1.4E-04
**TAG 52:2**	0.80	2.4E-05	0.83	9.9E-07	0.78	9.9E-08
**TAG 52:3**	0.86	1.9E-05	0.85	1.2E-05	0.84	6.0E-07
**TAG 52:4**	0.88	4.7E-04	0.88	9.9E-05	0.88	3.7E-05
**TAG 52:5**	0.83	5.8E-06	0.82	3.9E-05	0.86	5.5E-06
**TAG 52:6**	0.67	5.6E-05	0.73	1.6E-05	0.79	3.9E-06
**TAG 53:3**	0.81	3.2E-07	0.65	3.5E-05	0.79	4.7E-07
**TAG 53:4**	0.83	2.9E-05	0.78	7.7E-04	0.85	9.5E-06
**TAG 54:2**	0.83	3.4E-05	0.85	7.3E-04	0.82	6.7E-03
**TAG 54:3**	0.81	1.9E-05	0.83	2.6E-05	0.81	1.9E-06
**TAG 54:4**	0.89	4.8E-03	0.86	2.0E-04	0.83	1.7E-06
**TAG 54:5**	0.87	6.9E-05	0.85	7.1E-05	0.88	1.2E-04
**TAG 54:6**	n.s.		0.89	3.7E-03	n.s.	
**TAG 54:7**	0.85	3.5E-04	0.81	6.1E-06	0.90	1.4E-04
**TAG 56:4**	0.89	3.0E-03	0.87	3.9E-02	n.s.	
**TAG 56:5**	0.80	3.8E-04	0.80	2.7E-04	0.80	6.3E-06

Supplemental Table S2: Fatty acyl/alkyl composition of phospholipids in NIST SRM 1950, analyzed by direct infusion analysis in negative ion mode. Only species passing reproducibility quality control checks are listed.

Lipid class	Lipid species	Fatty acyl/ alkyl level
PC	PC 32:0	PC 16:0_16:0
	PC 32:2	PC 14:0_18:2
	PC 34:1	PC 16:0_18:1
	PC 34:2	PC 18:1_18:1
		PC 16:0_18:2
	PC 36:1	PC 18:0_18:1
		PC 16:0_20:1
	PC 36:2	PC 18:0_18:2
		PC 18:1_18:1
		PC 16:0_20:2
	PC 36:3	PC 18:1_18:2
		PC 16:0_20:3
		PC 18:0_18:3
	PC 36:4	PC 16:0_20:4
		PC 18:2_18:2
	PC 36:5	PC 16:0_20:5
	PC 37:4	PC 17:0_20:4
	PC 38:3	PC 18:0_20:3
		PC 18:2_20:1
	PC 38:4	PC 18:0_20:4
		PC 18:1_20:3
		PC 16:0_22:4
	PC 38:5	PC 18:1_20:4
		PC 16:0_22:5
		PC 18:2_20:3
	PC 38:6	PC 18:2_20:4
	PC 40:4	PC 18:0_22:4
	PC 40:5	PC 18:0_22:5
	PC 40:6	PC 18:0_22:6
	PC-O 36:4	PC O-16:0_20:4
	PC-O 36:5	PC O-16:1_20:4
	PC-O 38:4	PC O-18:0_20:4
	PC-O 38:5	PC O-18:1_20:4
	PC-O 38:6	PC O-18:2_20:4

Lipid class	Lipid species	Fatty acyl/ alkyl level
PE	PE 34:1	PE 16:0_18:1
	PE 34:2	PE 16:0_18:2
	PE 36:1	PE 18:0_18:1
	PE 36:2	PE 18:0_18:2
		PE 18:1_18:1
	PE 36:3	PE 18:1_18:2
		PE 16:0_20:3
	PE 36:4	PE 16:0_20:4
	PE 38:2	PE 18:0_20:2
	PE 38:3	PE 18:0_20:3
	PE 38:4	PE 18:0_20:4
		PE 16:0_22:4
	PE 38:5	PE 18:1_20:4
	PE 38:6	PE 16:0_22:6
	PE-O 36:3	PE O-18:1_18:2
	PE-O 36:5	PE O-16:1_20:4
	PE-O 38:5	PE O-18:1_20:4
	PE-O 38:6	PE O-18:2_20:4
	PE-O 38:7	PE O-16:1_22:6
	PE-O 40:5	PE O-20:1_20:4
	PE-O 40:6	PE O-18:1_22:5
PI	PI 34:2	PI 16:0_18:2
	PI 36:1	PI 18:1_18:0
	PI 36:2	PI 18:1_18:1
	PI 36:3	PI 16:0_20:3
	PI 36:4	PI 18:2_18:2
	PI 38:4	PI 18:0_20:4
	PI 38:5	PI 18:1_20:4

Supplemental Table S3: Fatty acyl composition of Cer, DG, and TG in NIST SRM 1950, analyzed by direct infusion analysis in positive ion mode. Only species passing reproducibility quality control checks are listed.

Lipid class	Lipid species	Fatty acyl level
**Cer**	Cer d42:1	Cer d18:1_24:0
**DAG**	DAG 34:1	DAG 16:0_18:1
	DAG 34:2	DAG 16:0_18:2
	DAG 36:2	DAG 18:1_18:1
	DAG 36:3	DAG 18:1_18:2
	DAG 36:4	DAG 18:2_18:2
**TG**	TG 46:2	TG 12:0_16:0_18:2
		TG 14:0_14:0_18:2
		TG 12:0_16:1_18:1
		TG 14:0_16:1_16:1
		TG 14:1_16:0_16:1
		TG 14:0_14:1_18:1
	TG 46:3	TG 10:0_18:1_18:2
		TG 14:0_14:1_18:2
		TG 12:1_16:0_18:2
		TG 12:1_16:1_18:1
		TG 14:1_16:1_16:1
		TG 14:1_14:1_18:1
		TG 14:0_14:0_18:3
	TG 48:0	TG 16:0_16:0_16:0
		TG 14:0_16:0_18:0
	TG 48:1	TG 16:0_16:0_16:1
	TG 48:2	TG 14:0_16:1_18:1
		TG 16:0_16:1_16:1
	TG 48:3	TG 16:1_16:1_16:1
		TG 14:1_16:1_18:1
		TG 14:0_16:0_18:3
	TG 50:2	TG 16:0_16:0_18:2
	TG 50:3	TG 16:1_16:1_18:1
	TG 50:5	TG 14:1_16:0_20:4
	TG 51:1	TG 15:0_17:1_19:0
	TG 51:2	TG 15:0_18:1_18:1
	TG 51:3	TG 16:0_17:1_18:2
		TG 17:1_17:1_17:1
		TG 15:1_17:0_19:2

Lipid class	Lipid species	Fatty acyl level
**TG**	TG 51:4	TG 15:0_18:2_18:2
		TG 15:0_18:1_18:3
	TG 52:4	TG 16:0_18:2_18:2
	TG 52:5	TG 16:0_16:1_20:4
	TG 52:6	TG 16:1_16:1_20:4
		TG 14:0_18:1_20:5
	TG 53:2	TG 17:1_17:1_19:0
	TG 53:3	TG 17:1_18:1_18:1
		TG 17:1_17:1_19:1
	TG 53:5	TG 17:1_18:2_18:2
		TG 17:1_17:1_19:3
		TG 17:1_18:1_18:3
		TG 17:3_18:1_18:1
		TG 17:0_17:3_19:2
	TG 54:2	TG 18:0_18:1_18:1
	TG 54:3	TG 18:1_18:1_18:1
		TG 16:0_18:2_20:1
	TG 54:6	TG 18:2_18:2_18:2
	TG 54:7	TG 18:2_18:2_18:3
		TG 18:1_18:3_18:3
		TG 16:1_18:1_20:5
		TG 16:1_16:1_22:5
	TG 56:4	TG 18:0_18:2_20:2
		TG 16:0_20:2_20:2
		TG 18:2_18:2_20:0
	TG 56:5	TG 18:1_18:1_20:3
		TG 18:2_18:2_20:1
	TG 56:6	TG 18:1_18:1_20:4
		TG 16:0_18:0_22:6
	TG 56:8	TG 16:0_20:4_20:4
		TG 18:1_18:3_20:4
		TG 16:0_18:3_22:5
		TG 18:1_18:1_20:6
	TG 58:6	TG 18:1_18:1_22:4
	TG 58:8	TG 18:2_20:3_20:3

Supplemental Table S4: Sample run order in this study. Chromatographic runs (RP and HILIC) additionally included ten injections of a QC sample at the beginning for equilibration of the chromatographic system.

sample name (running order)	Sample type
001_QC<br>002_QC	**QC**
003_blank<br>004_blank<br>005_blank<br>006_blank	**blank**
007_QC<br>008_QC	**QC**
009_quant_LTR<br>010_quant_LTR<br>011_quant_LTR<br>012_quant_LTR<br>013_quant_LTR	**LTR**
014_QC<br>015_QC	**QC**
016_quant_NIST<br>017_quant_NIST<br>018_quant_NIST<br>019_quant_NIST<br>020_quant_NIST	**NIST**
021_QC<br>022_QC	**QC**
023_lin0<br>024_lin10<br>025_lin25<br>026_lin50<br>027_lin75<br>028_lin100<br>030_lin150<br>032_lin200<br>033_lin200<br>035_lin150<br>037_lin100<br>038_lin75<br>039_lin50<br>040_lin25<br>041_lin10<br>042_lin0	**Linearity**
043_QC<br>044_QC	**QC**

Supplemental Table S5: An example of a typical analysis worklist that includes conditioning QCs to stabilize the system, replicate extracted blanks to check the presence of contaminants and carryover, dilutions of a QC sample to evaluate linearity replicates of standard reference material for normalization and Pooled QC samples (typically every 10 study samples) for batch correction (Adapted from [1]).

Injection number	Sample Type
00	System conditioning QCs
1	Extracted blank
2	Extracted blank
**3**	**Standard reference sample (NIST or long term reference)**
**4**	**Standard reference sample (NIST or long term reference)**
5	Pooled QC
6	Dilution series
7	Dilution series
8	Dilution series
9	Dilution series
10	Dilution series
11	Pooled QC
12	Study sample 1
13	Study sample 2
14	Study sample 3
…	…
n–11	Pooled QC
n–10	Dilution series
n–9	Dilution series
n–8	Dilution series
n–7	Dilution series
n–6	Dilution series
n–5	Pooled QC
**n–4**	**Standard reference sample (NIST or long term reference)**
**n–3**	**Standard reference sample (NIST or long term reference)**
n–2	Extracted blank
n–1	Extracted blank
n	Pooled QC

[1] Broadhurst, D., R. Goodacre, S. N. Reinke, J. Kuligowski, I. D. Wilson, M. R. Lewis, and W. B. Dunn. 2018. Guidelines and considerations for the use of system suitability and quality control samples in mass spectrometry assays applied in untargeted clinical metabolomic studies. Metabolomics. 14: 72. [online] https://doi.org/10.1007/s11306-018-1367-3.

SUPPLEMENTAL FIGURES

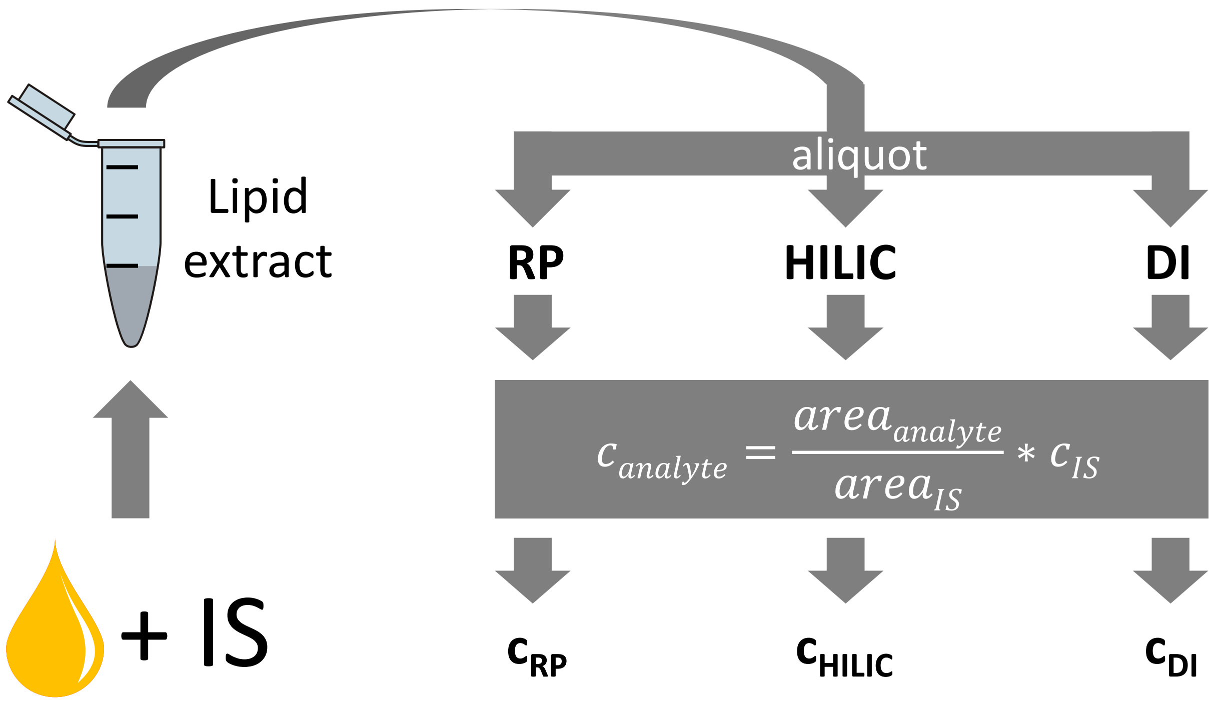

Supplemental Figure S1: Sample preparation scheme. Plasma was extracted in the presence of internal standards, and aliquots of lipid extracts were split for analysis with RP/HILIC/DI and HRMS detection. Data analysis, normalization to class-specific internal standards, quality control filtering, and calculation of lipid concentrations was performed separately for each method.

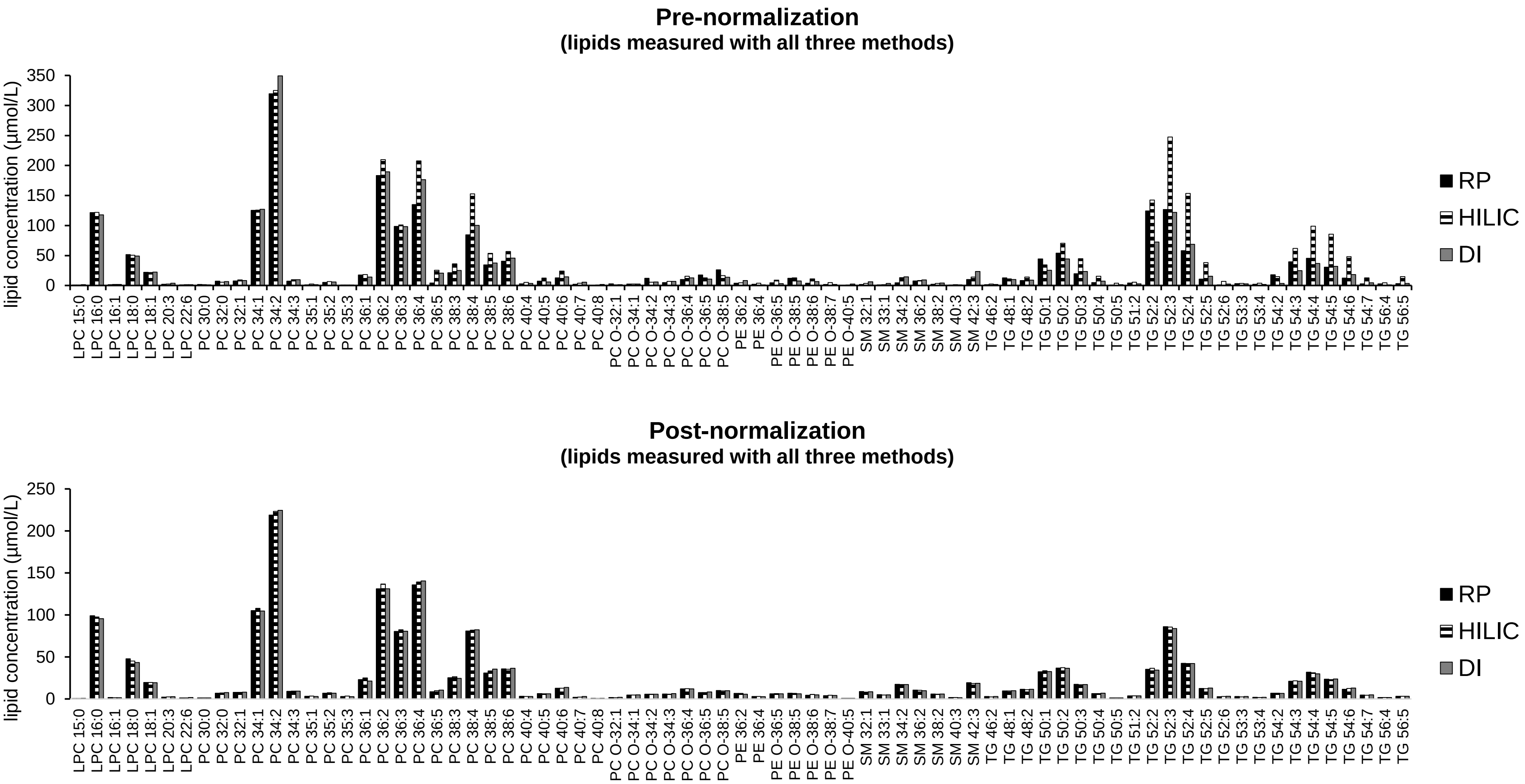

Supplemental Figure S2: Total calculated lipid concentrations of the same sample measured with three different sample introduction methods and comparable mass spectrometric detection, before (top panel) and after (lower panel) normalization to a standard reference sample.75 lipids detected with all three methods, and for which NIST consensus values are available, are shown.

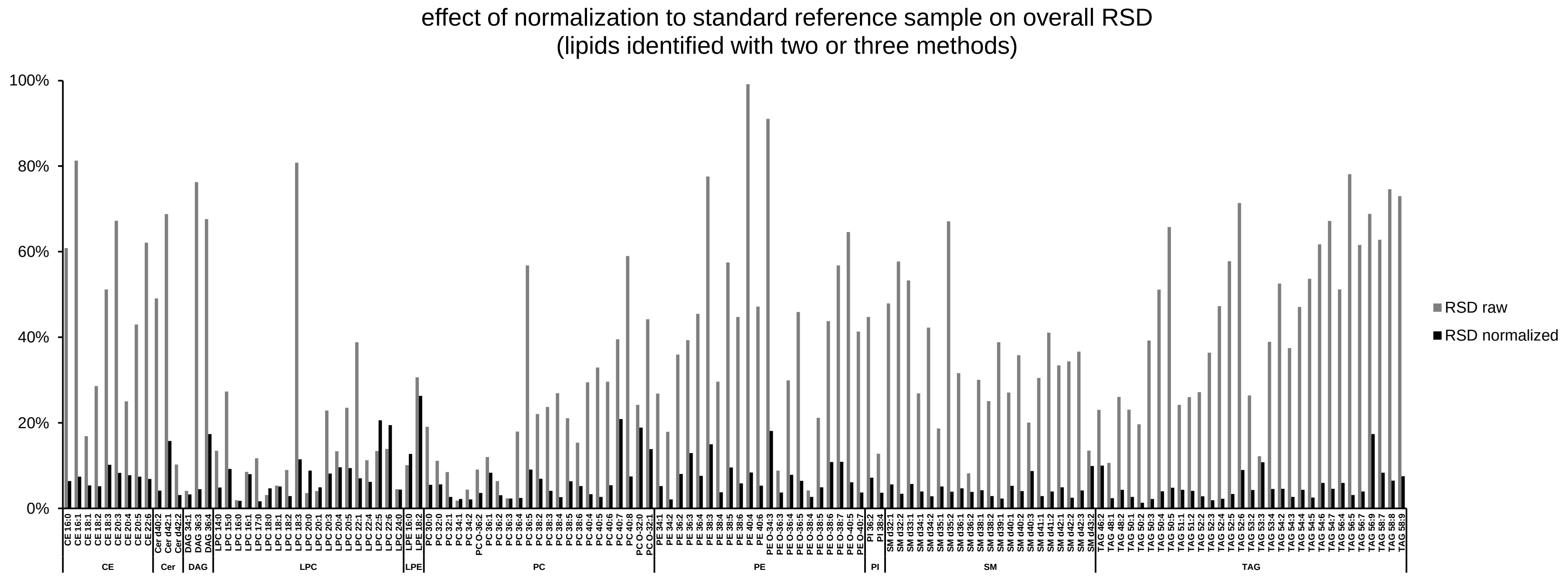

Supplemental Figure S3: Variability of lipid concentrations when measuring the same samples with different sample introduction methods (RP, HILIC, DI), before and after normalization, represented as total CoV (coefficient of variation). Small value indicates good agreement between concentrations measured by different methods, large CoV indicates large differences between concentration values measured with the different methods. Only lipids measured with more than one sample introduction method are shown.

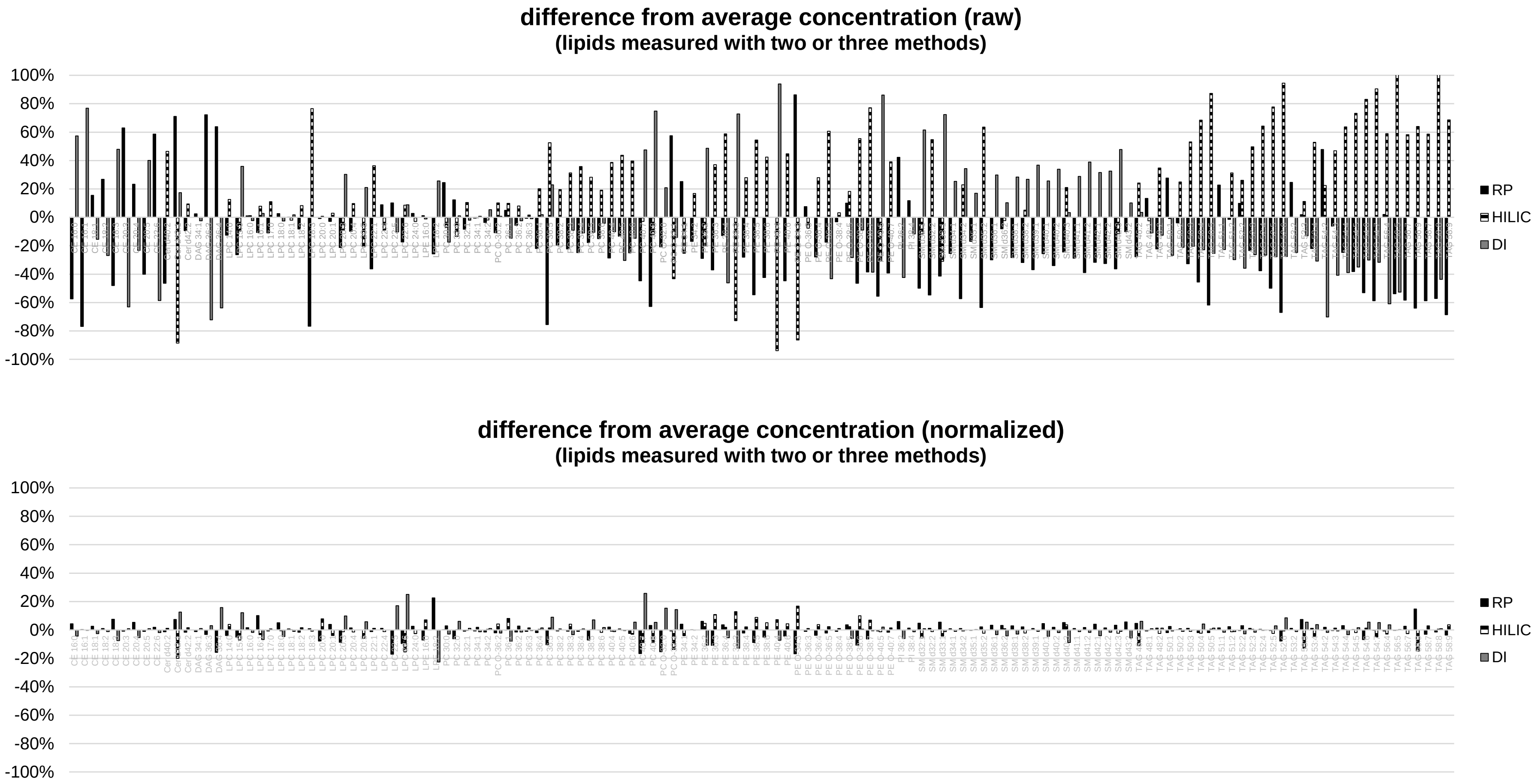

Supplemental Figure S4: Normalization to a standard reference material removes method-dependent quantitative bias, represented as difference from average concentration between the three methods. Identical y-axis scaling was used in both figures.

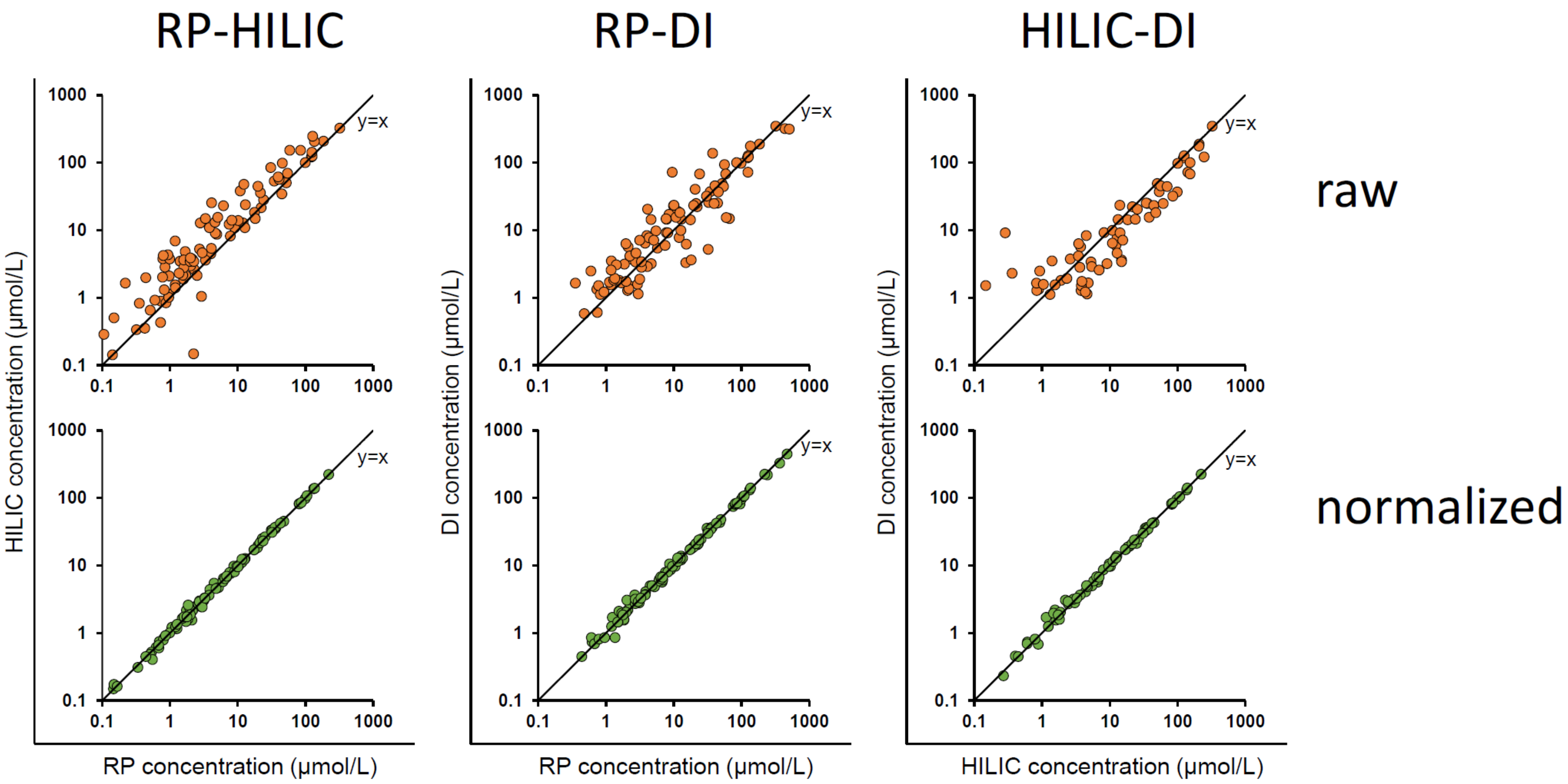

Supplemental Figure S5: Correlation between lipid concentrations measured in plasma with three different sample introduction methods, before (top) and after (bottom) normalization to a standard reference sample. Almost no quantitative bias is present between the concentration values obtained with RP, HILIC, or DI after normalization.

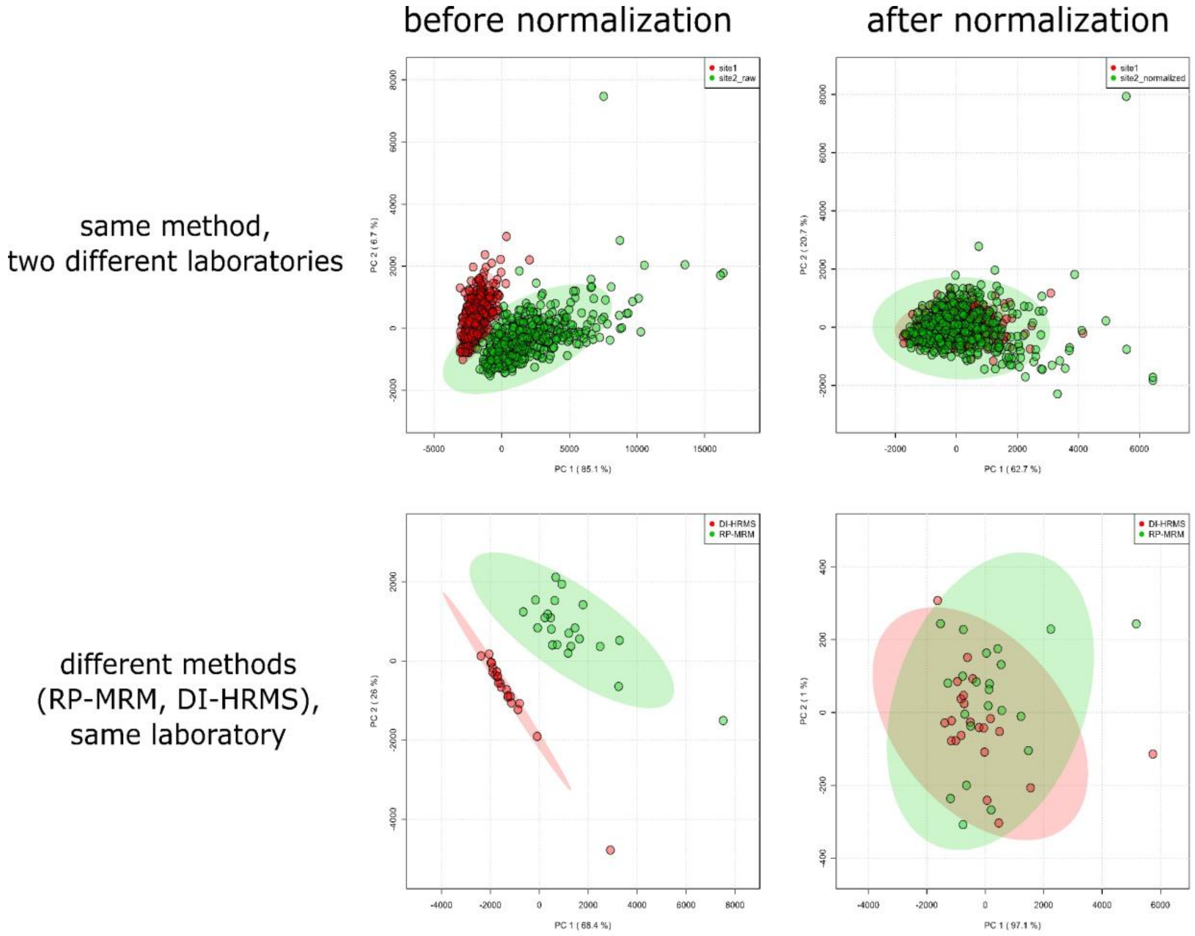

Supplemental Figure S6: PCA plots showing how inter-site and inter-method variability can be removed to a large extent by normalizing to a common reference sample. Top row shows data from an inter-laboratory study, where the same sample set (478 plasma samples) was analyzed with the same RP-MRM method in two different laboratories. The bottom row shows data from the same lipid extracts measured with a DI-HRMS method and with an RP-MRM method. In both cases, normalization to a common reference sample improves comparability and removes method-dependent quantitative differences.

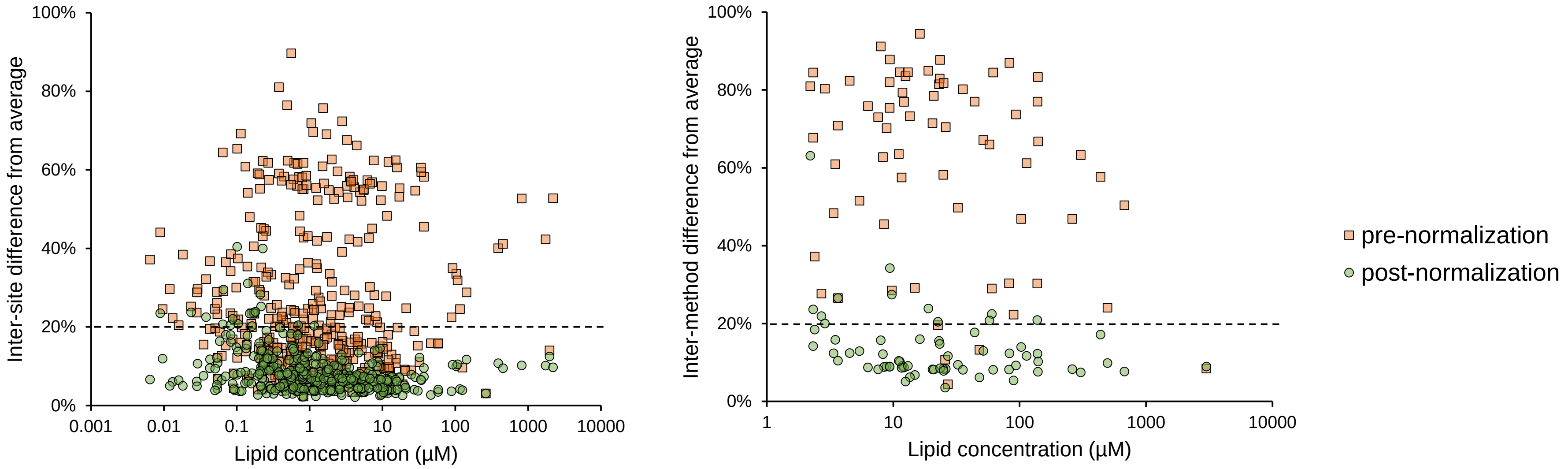

Supplemental Figure S7: normalization to a standard reference sample drastically improves method comparability, represented as relative difference from the mean of the same samples measured with either the same RP-MRM method in two different laboratories (left), or with both a RP-MRM and a DI-HRMS method in the same site (right).
